# The complete chloroplast genome sequence of *Styrax agrestis* (Lour.) G. Don (Styracaceae)

**DOI:** 10.1080/23802359.2021.1882351

**Published:** 2021-03-11

**Authors:** Hongchao Wang, Yaoqin Zhang, Lili Tong, Yukun Tian, Xiaoyu Jiang, Xiaogang Xu

**Affiliations:** a Co-Innovation Center for Sustainable Forestry in Southern China, College of Biology and the Environment, Key Laboratory of State Forestry and Grassland Administration on Subtropical Forest Biodiversity Conservation, Nanjing Forestry University, Nanjing, China; b State Environmental Protection Scientific Observation and Research Station for Ecology and Environment of Wuyi Mountains, Nanping, China; c School of Horticulture and Landscape Architecture, Jinling Institute of Technology, Nanjing, China

**Keywords:** *Styrax agrestis*, phylogenomics, Styracaceae, complete chloroplast genome

## Abstract

*Styrax agrestis* (Lour.) G. Don, is a deciduous species of Styracaceae with beautiful shape, drooping flowers, and blooming like snow. Here, we characterized the complete chloroplast (cp) genome of *S. agrestis* using next generation sequencing. The circular complete cp genome of *S. agrestis* is 157,893 bp in length, containing a large single-copy (LSC) region of 87,512 bp, and a small single-copy (SSC) region of 18,285 bp. It comprises 136 genes, including eight rRNA genes, 37 tRNAs genes, 90 protein-coding genes, and one pseudo gene. The GC content of *S. agrestis* cp genome is 36.96%. The phylogenetic analysis suggests that *S. agrestis* is a sister species to *Styrax faberi* in Styracaceae.

*Styrax agrestis* (Lour.) G. Don is mainly distributed in tropical and subtropical lowland areas in Southeast Asia. Currently, *S*. *agrestis* possesses high value for ornamental, timber, and medicinal purposes (Huang and Grimes 2003). So far, there is still no complete cp genome characterized for *S*. *agrestis*. Here, we characterized the complete cp genome sequence of *S*. *agrestis* (GenBank accession number: MT644192) based on Illumina pair-end sequencing to provide a valuable complete cp genomic resource.

Total genomic DNA was isolated from fresh leaves of S. *agrestis* grown in Jianfengling, Ledong County (N 18.4210, E 108.5055), Hainan, China. The voucher specimen was deposited at the herbarium of Nanjing Forestry University (accession number: NF2020090). The whole genome sequencing was carried out on Illumina Hiseq platform by Nanjing Genepioneer Biotechnology Inc. (Nanjing, China). The original reads were filtered by CLC Genomics Workbench v9, and the clean reading was assembled into chloroplast (cp) genome with SPAdes (Bankevich et al. 2012). Finally, CpGAVAS (Liu et al. 2012) was used to annotate the gene structure and OGDRAW (Lohse et al. 2013) was used to generate the physical map. Based on the maximum likelihood (ML), the phylogenetic tree was deduced by MAFFT (Rozewicki et al. 2019) and MEGA version 7 (Kumar et al. 2016).

The circular genome of *S. agrestis* was 157,893 bp in size and contained two inverted repeat (IRa and IRb) regions of 26,048 bp, which were separated by a large single-copy (LSC) region of 87,512 bp, and a small single-copy (SSC) region of 18,285 bp. A total of 136 genes are encoded, including 90 protein-coding genes (82 CDS species), 37 tRNAs gene (30 tRNA species), eight rRNA genes (four rRNA species), and one pseudo gene. Most of genes occurred in a single copy; however, eight protein-coding genes (*ndhB*, *orf42, rpl2, rpl23, rps12, rps7, ycf2*, and *ycf15*), seven tRNA genes (*trnA-UGC, trnI-CAU, trnI-GAU, trnL-CAA, trnN-GUU, trnR-ACG*, and *trnV-GAC*), and four rRNA genes (*4.5S, 5S, 16S*, and *23S*) are totally duplicated. A total of nine protein-coding genes (*atpF, ndhA, ndhB, petB, petD, rpl16, rpoC1, rps16*, and *rpl2*) contained one intron while the other three genes (*clpP, ycf3, rps12*) had two intron each. The rest of the 70 protein-coding genes are *psaA, psaB, psaC, psaI, psaJ, psbA, psbB, psbC, psbD, psbE, psbF, psbH, psbI, psbJ, psbK, psbL, psbM, psbN, psbT, ndhC, ndhD, ndhE, ndhF, ndhG, ndhH, ndhI, ndhJ, ndhK, petA, petG, petL, petN, atpA, atpB, atpE, atpH, atpI, rbcL, rpl14, rpl20, rpl22, rpl23, rpl32, rpl33, rpl36, rps11, rps14, rps15, rps18, rps19, rps2, rps3, rps4, rps7, rps8, rpoA, rpoB, rpoC2, matK, cemA, accD, ccsA, infA, lhbA, orf188, orf42, ycf1, ycf15, ycf2*, and *ycf4.* The overall GC content of *S. agrestis* genome is 36.96%, and the corresponding values in LSC, SSC, and IR regions are 34.81%, 30.29%, and 42.92%, respectively.

The phylogenetic analysis was conducted based on 35 Styracaceae cp genomes and three taxa (Symplocaceae, Ebenaceae, and Clethraceae) as outgroups with sequenced cp genomes. We found that *S. agrestis* was clustered with other families of Styracaceae with 100% bootstrap values (Figure 1). In addition, *S. agrestis* was highly supported to be a sister species to *Styrax faberi* in Styracaceae.

**Figure 1.**
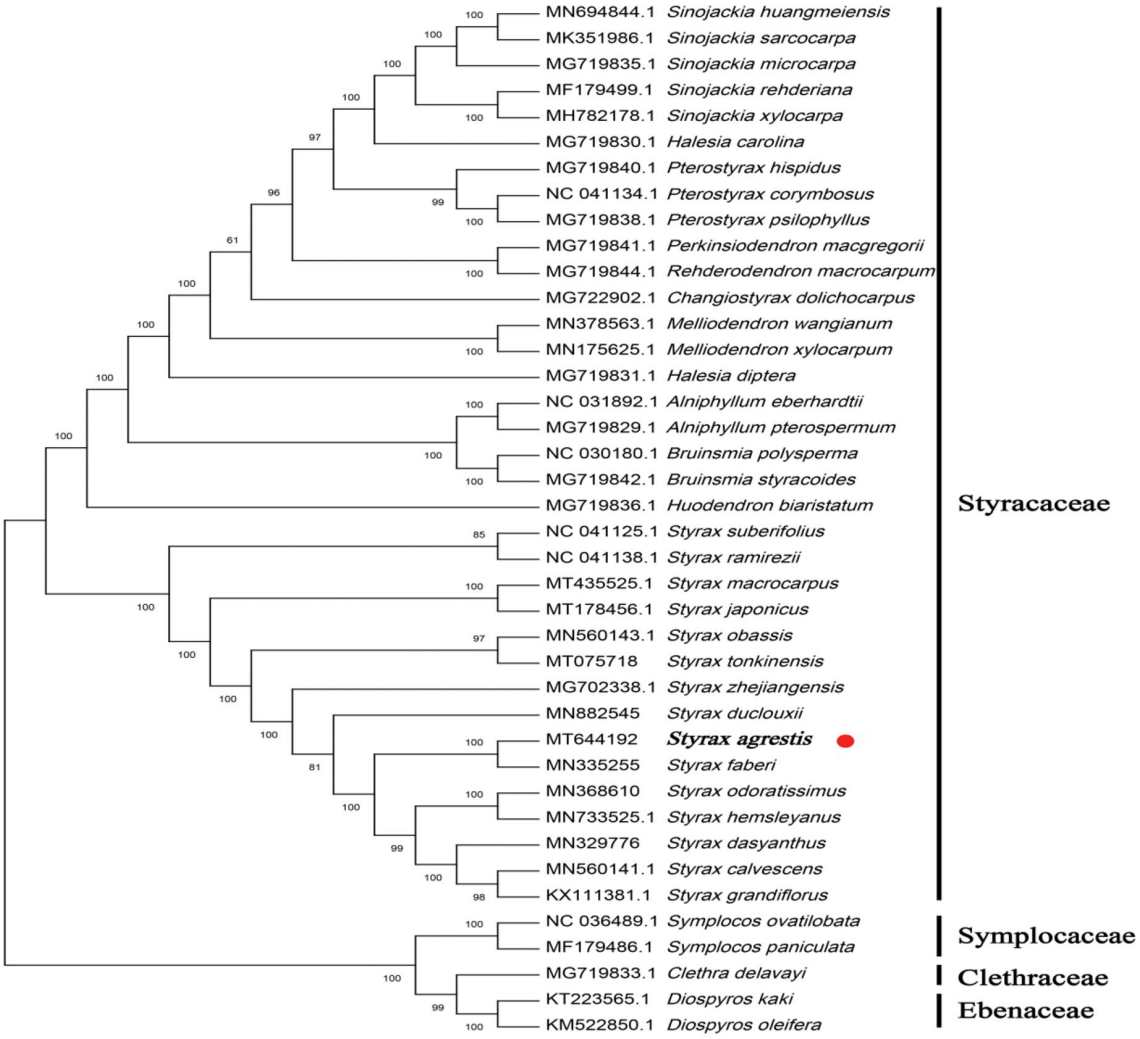
Maximum-likelihood tree showing the relationship among *Styrax agrestis* and representative species within Styracaceae, based on whole chloroplast genome sequences, with three taxa from Ericales as outgroup. The bootstrap supports the values shown on the branches.

## Data Availability

The raw sequence data are accessible from: https://pan.baidu.com/s/15eih-q4yDMD4sjosDLo7yA (password: acvm) the GenBank accession number: MT644192.
